# Bilateral simultaneous hip and knee replacement: an epidemiological nationwide study from 2001 to 2016

**DOI:** 10.1186/s12893-024-02450-y

**Published:** 2024-05-31

**Authors:** Umile Giuseppe Longo, Rocco Papalia, Alessandro Mazzola, Stefano Campi, Sergio De Salvatore, Vincenzo Candela, Andrea Vaiano, Ilaria Piergentili, Vincenzo Denaro

**Affiliations:** 1 Fondazione Policlinico Universitario Campus Bio-Medico, Via Alvaro del Portillo, Roma, 200 - 00128 Italy; 2 Research Unit of Orthopaedic and Trauma Surgery, Department of Medicine and Surgery, Università Campus Bio- Medico di Roma, Via Alvaro del Portillo, Roma, 21 - 00128 Italy; 3 Department of Statistical Sciences, Sapienza University of Rome, Piazzale Aldo Moro, 5, Roma, 00185 RM Italy; 4 Orthopedic Unit, Department of Surgery, Bambino Gesù Children’s Hospital, Rome, Italy

**Keywords:** Hip, Knee, Joint, Replacement, Bilateral, Simultaneous, Epidemiology, Demographic

## Abstract

**Background:**

Several studies have compared the pros and cons of simultaneous bilateral versus staged bilateral hip and knee replacement but the outcomes of these two surgical options remains a matter of controversy. This study aimed to evaluate demographic features, incidence and hospitalization rates of bilateral one stage total hip and knee arthroplasty in Italy.

**Methods:**

The Italian Ministry of Health’s National Hospital Discharge Reports (SDO) were used to gather data. This study referred to the adult population (+ 20 years of age) from 2001 to 2015 for hip arthroplasty and from 2001 to 2016 for knee arthroplasty.

**Results:**

Overall, 1,544 bilateral simultaneous hip replacement were carried out. The incidence rate was 0.21 cases per 100,000 adult Italian residents. Male/female ratio was 1.1. The average days of hospital stay was 11.7 ± 11.8 days. The main primary codified diagnosis was: osteoarthrosis, localized, primary, pelvic region and thigh (ICD code: 715.15). 2,851 bilateral simultaneous knee replacement were carried out. The incidence rate was 0.37 cases per 100,000 adult Italian residents. Male/female ratio was 0.6. The average days of hospital stay was 7.7 ± 5.8 days. The main primary codified diagnosis was: osteoarthrosis, localized, primary, lower leg (ICD code: 715.16).

**Conclusions:**

The burden of hip and knee osteoarthrosis as a leading cause of bilateral joint replacement is significant in Italy. The national registers’ longitudinal analysis may provide data for establishing international guidelines regarding the appropriate indications for one stage bilateral simultaneous hip or knee replacement versus two stage.

## Background

Patients with severe arthropathies can benefit from total joint arthroplasty (TJA), which includes total knee arthroplasty (TKA) and total hip arthroplasty (THA) such as osteoarthritis (OA), which can greatly ameliorate these patients’ quality of life [1–5]. As life expectancy increases, it is expected that the prevalence of arthropathies continues to rise in parallel worldwide [6, 7]. Undoubtedly, arthroplasty procedures will put a tremendous financial strain on the public health-care system with the passing of time [6]. Bilateral THA or TKA can be performed as two staged unilateral arthroplasties under separate anesthetics and hospitalizations or as a one stage bilateral surgery under the same anesthetic and during one hospitalization. Bilateral one stage THA was firstly described by Jaffe and Charnley more than 50 years ago [8]. Historically, the most frequent causes of such extensive involvement of multiple joints have been OA and rheumatoid arthritis (RA) [1, 9, 10]. However, several studies have compared the pros and cons of simultaneous bilateral versus staged bilateral TKA and THA but the outcomes of these two surgical options remains a matter of controversy [11–15]. In general, studies have shown superior clinical results for simultaneous bilateral TKA and THA. Simultaneous TKA showed higher prosthesis survival rates in 10-year [16], shorter hospitalizations [14] and lower frequency of periprosthetic joint infections rates [17, 18] when compared to staged TKA. Similarly, simultaneous THA has been linked to lower rates of deep vein thrombosis and pulmonary embolism [15], reduced blood loss [19], with no appreciable variations in mortality or hip dislocation rates [15].

National registries constitute the most appropriate tool for evaluating epidemiological data [20]. Unfortunately, despite the fact that numerous studies have examined the postoperative outcomes, in the available literature there are few studies regarding demographic features, incidence and trends of hospitalization of patients undergoing bilateral one stage TJA in the Italian population.

The purpose of this study was to evaluate demographic features, hospitalization trends and incidence of bilateral simultaneous THA from 2001 to 2015 and TKA from 2001 to 2016 in Italy. The longitudinal examination of national registers may be helpful to obtain these data: they are crucial to provide data for establishing international guidelines regarding the appropriate indications for one stage bilateral simultaneous THA or TKA versus two stage.

## Methods

Data from 2001 to 2015 for bilateral simultaneous THA and from 2001 to 2016 for bilateral simultaneous TKA were supplied by the Italian Ministry of Health in the National Hospital Discharge records (SDO) archive. Patients’data about age, sex, length of hospital stay, diagnosis and procedure codes were collected. The annual adult population size was provided by the National Institute for Statistics (ISTAT). In order to group patients with bilateral one stage THA and TKA, we selected cases with the International Classification of Diseases, Ninth Revision, Clinical Modification (ICD-9-CM) codes 81.51 or 81.54 in both primary or secondary procedures. We included data only from patients who underwent a procedure (THA or TKA) classified as bilateral according to the (ICD-9-CM). Only adult patients were involved in the study, therefore people aged at least 20 years. All methods were performed in accordance with the relevant guidelines and regulations.

### Statistics

Descriptive statistical analyses were performed. For categorical variables, frequencies and percentages were calculated. For continuous variables, means and standard deviations were used. Incidence was determined as the ratio between the number of cases and the size of the adult population, referring to 100,000 inhabitants (cases/population*100,000). All statistical analyses were carried out with the IBM SPSS Statistics for Windows, Version 26.0. (Armonk, NY: IBM Corp) and Microsoft Excel (2019).

## Results

### Bilateral THA

#### Demographics

**Fig. 1:**
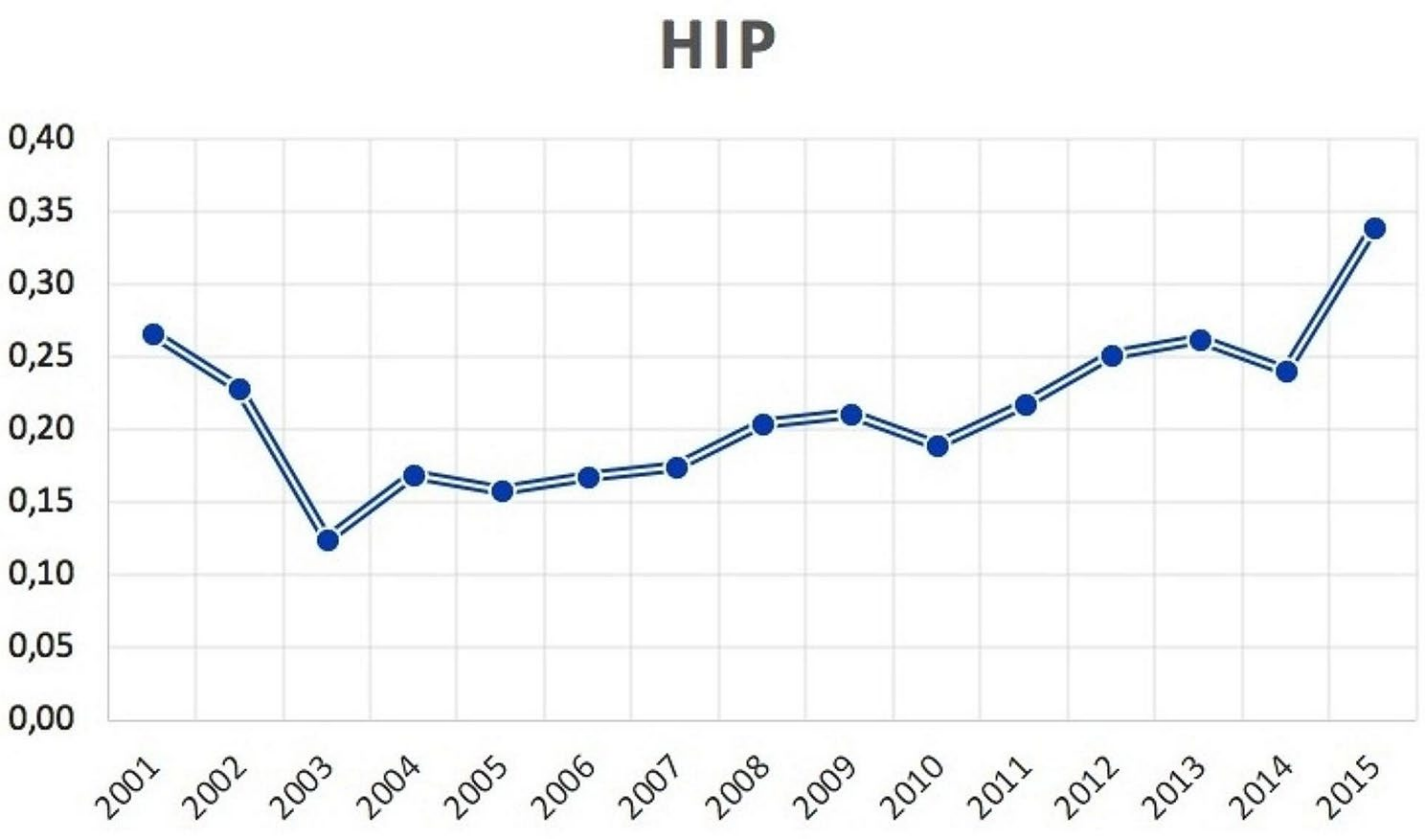
Incidence of bilateral simultaneous THA per 100,000 residents by 2001 to 2015 in Italy

**Fig. 2:**
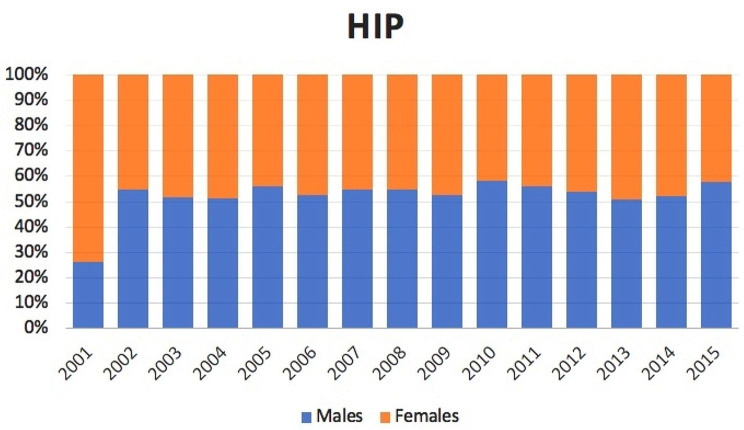
Bilateral simultaneous THA in the study period stratified for gender

1,544 bilateral THA were performed between 2001 and 2015 in the adult population. The number of bilateral THA decreased from 2001 (122 cases) to 2003 (58 cases), while increased from 2003 to 2015 (168 cases). The cumulate period of incidence was 0.21 cases of bilateral THA for every 100,000 Italian adult residents. The incidence rate increased from 0.27 for every 100,000 residents in 2001 to 0.34 for every 100,000 residents in 2015 (Fig. 1). Male/female ratio was 1.1 (from 0.4 in 2001 to 1.4 in 2015). Overall, 48% of patients were females. Females represented the majority of patients who underwent bilateral THA only in the 2001 year (Fig. 2).

**Fig. 3:**
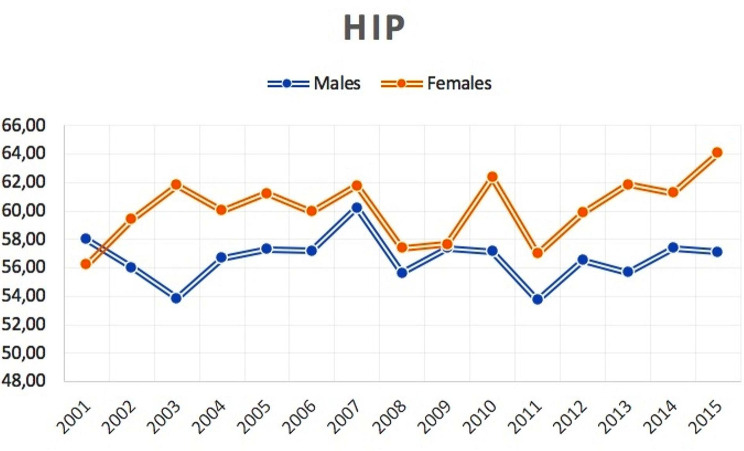
Average age of male and female patients undergoing bilateral simultaneous THA over the study period

The average age of patients was 58.3 ± 12.6 (56.7 ± 11.7 years for males and 60 ± 13.4 years for females). Except in 2001, female always had a higher average age than males (Fig. 3).

#### Hospitalization length

**Fig. 4:**
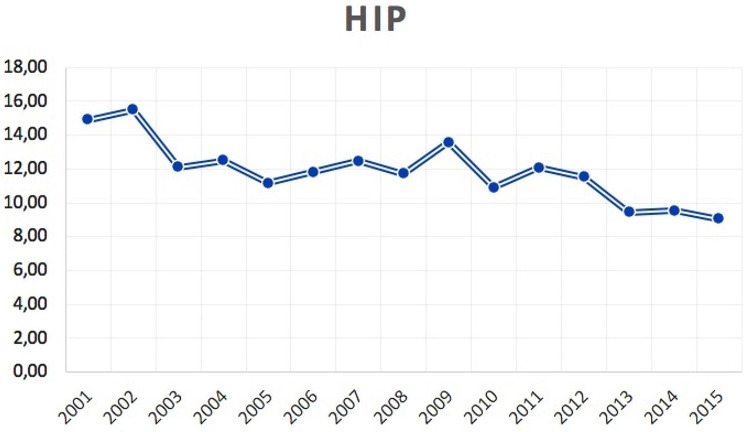
Mean days of hospital stay for patients undergone bilateral simultaneous THA from 2001 to 2015

The average days of hospital stay was 11.7 ± 11.8 days, from a minimum of 1 to a maximum of 157 days (males 10.7 ± 10.7 days and females 12.9 ± 12.8 days). The trend of the mean days of hospital stay from 2001 to 2015 was decreasing (Fig. 4).

#### Main primary diagnoses

**Fig. 5:**
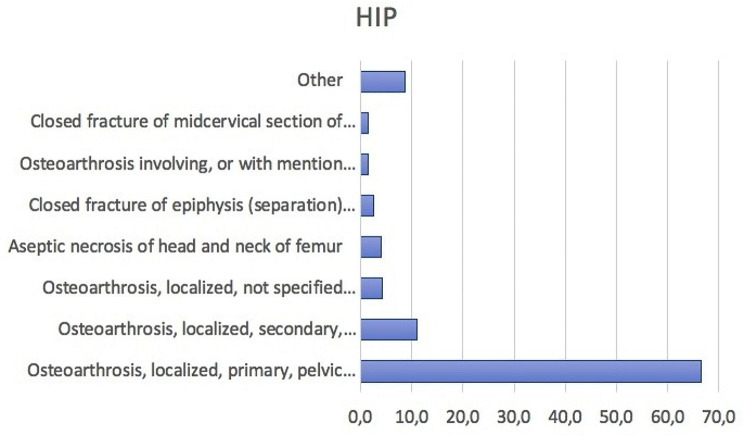
Main primary diagnoses (according to the ICD-9-CM) requiring bilateral simultaneous THA from 2001 to 2015

The main primary diagnoses most frequently made over the 15-year study period were: Osteoarthrosis, localized, primary, pelvic region and thigh (ICD code: 715.15, 66.5%); Osteoarthrosis, localized, secondary, pelvic region and thigh (ICD code: 715.25, 11.1%); Osteoarthrosis, localized, not specified whether primary or secondary, pelvic region and thigh (ICD code: 715.35, 4.3%); Aseptic necrosis of head and neck of femur (ICD code: 733.42, 4.0%); Closed fracture of epiphysis (separation) (upper) of neck of femur (ICD code: 820.01, 2.5%); Osteoarthrosis involving, or with mention of more than one site, but not specified as generalized, site unspecified (ICD code: 71,580, 1.5%); Closed fracture of midcervical section of neck of femur (ICD code: 82,002, 1.4%) (Fig. 5).

### Bilateral TKA

#### Demographics

**Fig. 6:**
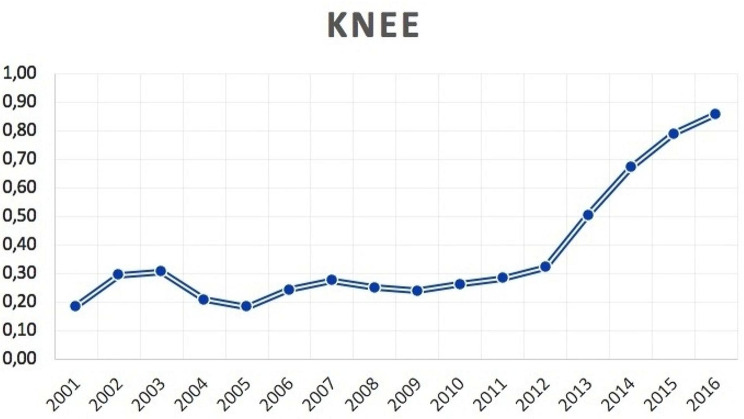
Incidence of bilateral simultaneous TKA per 100,000 residents by 2001 to 2016 in Italy

**Fig. 7:**
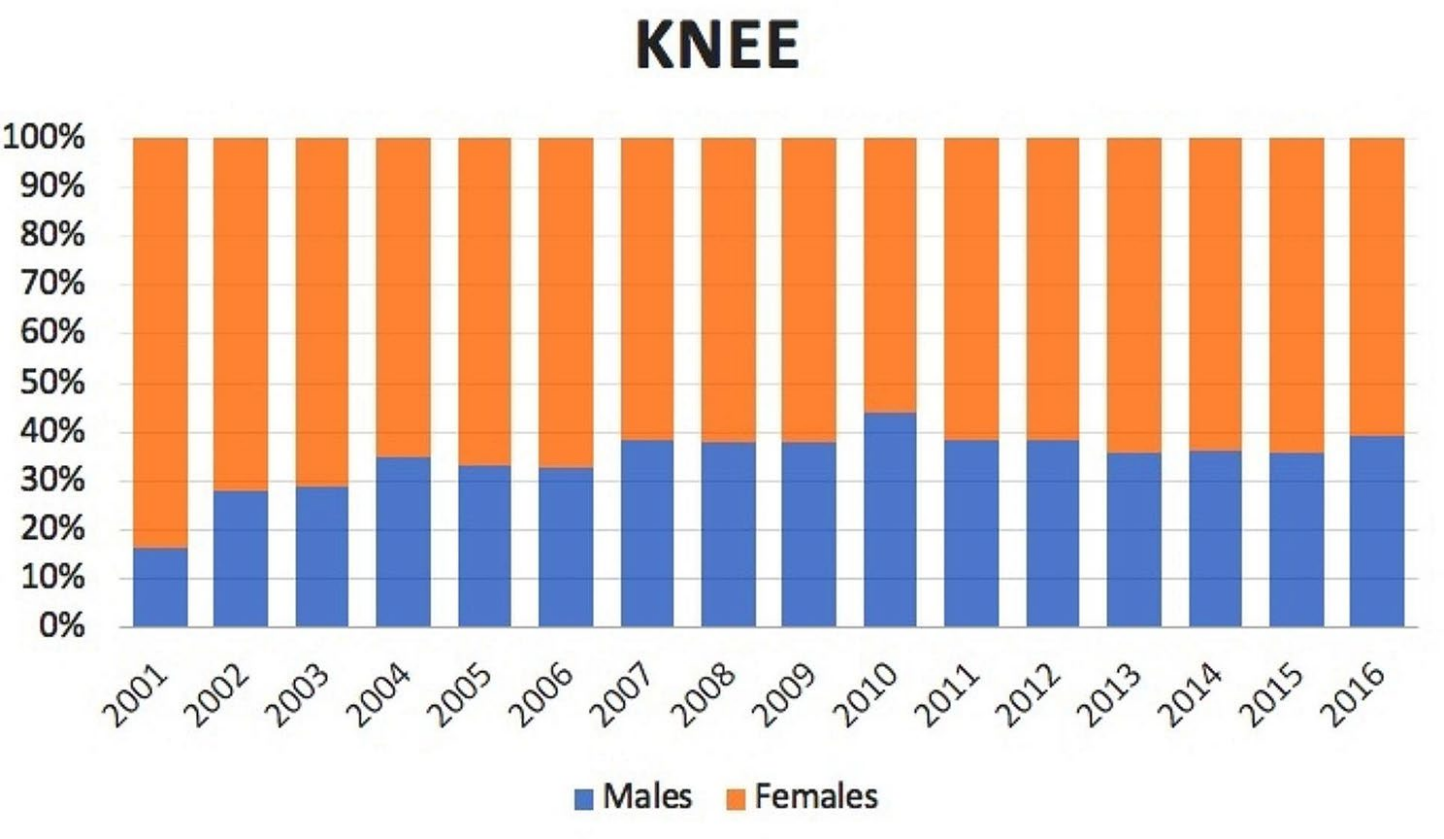
Bilateral simultaneous TKA in the study period stratified for gender

**Fig. 8:**
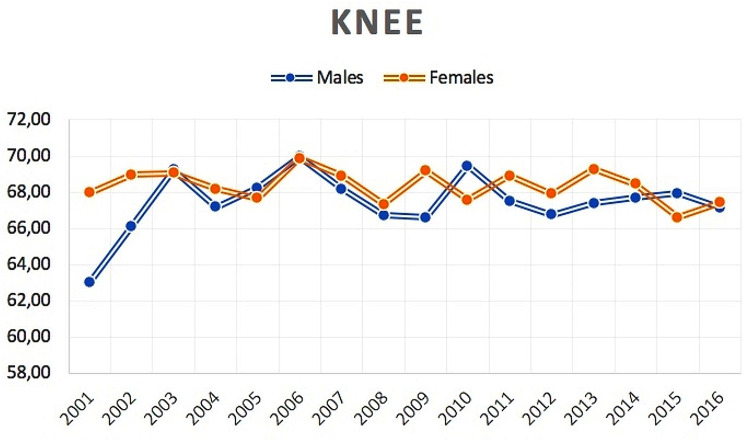
Average age of male and female patients undergoing bilateral simultaneous TKA over the study period

2,851 bilateral TKA were performed from 2001 to 2016 in the adult population. The number of bilateral TKA increased from 85 cases in 2001 to 425 cases in 2016. The cumulate period of incidence was 0.37 cases of bilateral TKA for every 100,000 Italian adult residents. The incidence rate increased from 0.19 for every 100,000 residents in 2001 to 0.86 for every 100,000 residents in 2016 (Fig. 6). Male/female ratio was 0.6 (from 0.2 in 2001 to 0.6 in 2016). Overall, 64.3% of patients were females. Females represented at each year the majority of patients who underwent bilateral TKA (Fig. 7). The patients’average age was 68 ± 8.3 (67.6 ± 8.7 years males and 68.2 ± 8.1 years females) (Fig. 8).

#### Length of hospital stay

**Fig. 9:**
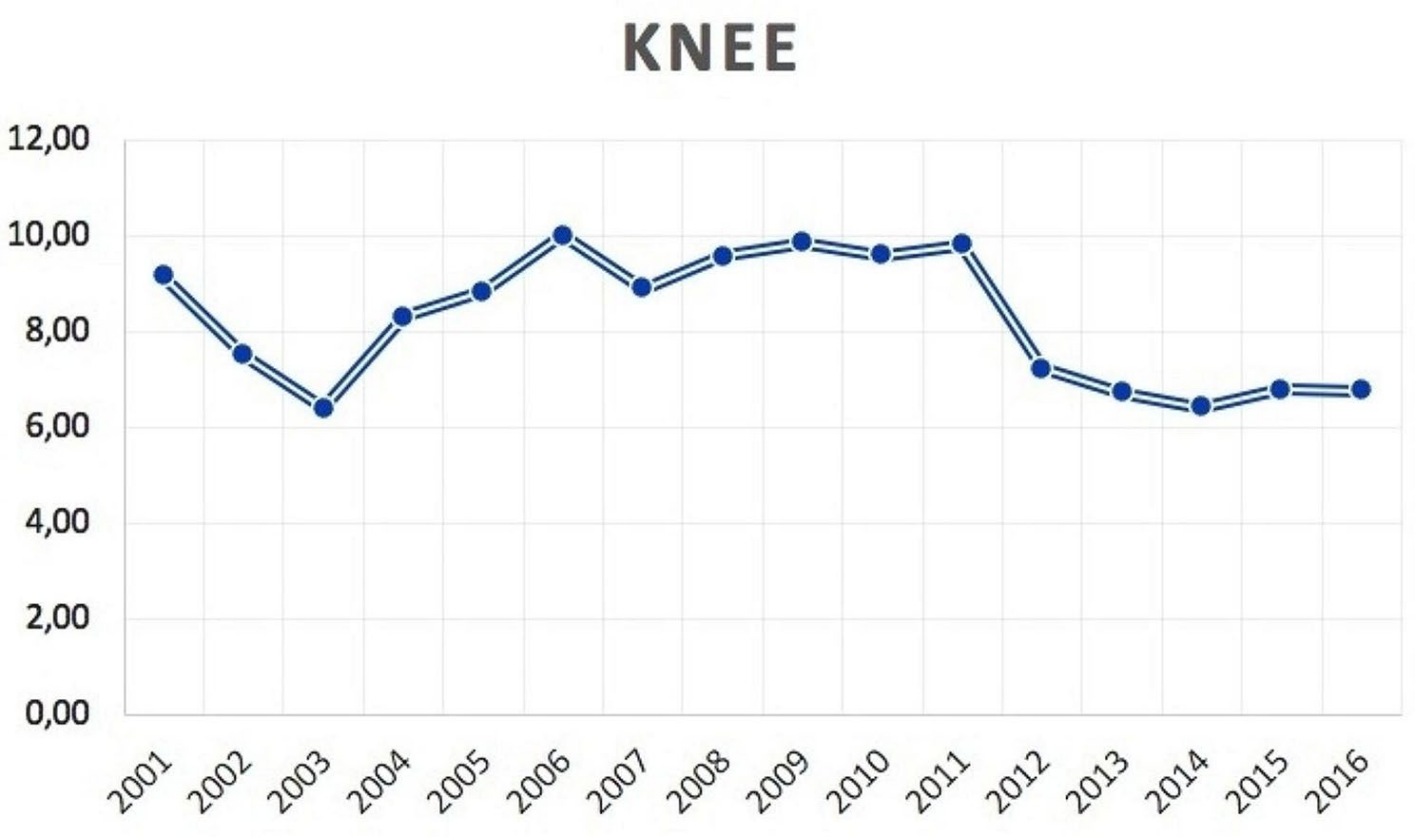
Mean days of hospital stay for patients undergone bilateral simultaneous TKA from 2001 to 2016

The average days of hospital stay was 7.7 ± 5.8 days, from a minimum of 1 to a maximum of 93 days (males 7.7 ± 5.9 days and females 7.7 ± 5.8 days). The trend of the mean days of hospital stays from 2001 to 2015 was decreasing (Fig. 9).

#### Main primary diagnoses

**Fig. 10:**
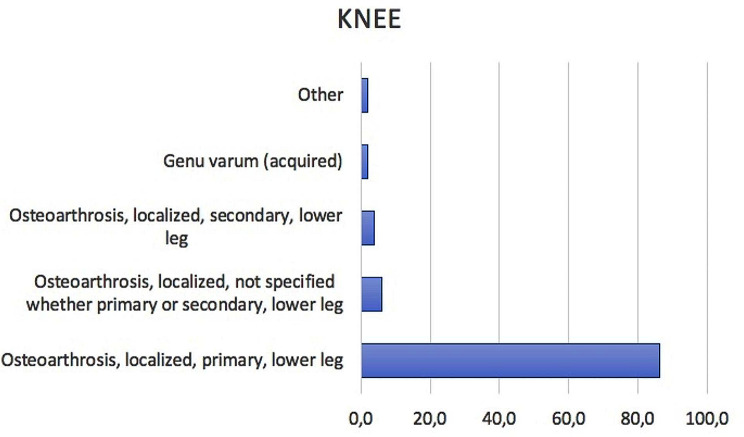
Main primary diagnoses (according to the ICD-9-CM) requiring bilateral simultaneous TKA from 2001 to 2016

Over the study period, main primary diagnoses were: Osteoarthrosis, localized, primary, lower leg (ICD code: 715.16, 86.2%); Osteoarthrosis, localized, not specified whether primary or secondary, lower leg (ICD code: 715.36, 6.0%); Osteoarthrosis, localized, secondary, lower leg (ICD code: 715.26, 4.0%); Genu varum (acquired) (ICD code: 736.42, 1.9%) (Fig. 10).

## Discussion

Relieving pain and regaining function are two of total joint arthroplasty’s primary objectives. In the present study, bilateral one stage THA showed an incidence of 0.21 cases for every 100,000 Italian adult residents, whereas bilateral one stage TKA showed an incidence of 0.37 cases for every 100,000 Italian adult residents. Except for the 2001–2003 period, the present study highlighted an increasing incidence rate of one stage bilateral THA and TKA in Italy. Several studies have tried to investigate the pros and cons of simultaneous bilateral versus staged bilateral TKA and THA. Literature shows that performing one stage bilateral TKA provides a number of benefits, including a single anesthetic exposure [12], a single postoperative course of pain and rehabilitation period [13], decreased overall hospitalization, more effective resource use, and higher patient satisfaction [14]. A systematic review and meta-analysis comparing simultaneous bilateral THA versus staged bilateral THA revealed significantly lower rates of deep vein thrombosis, pulmonary embolism and respiratory problems in the first group, with no appreciable variations in mortality or hip dislocation rates [15]. However, when performing a TKA, surgical procedures including tourniquet inflation and deflation, cementing, and bone preparation may increase the risk of vascular, respiratory, hematological and neurological alterations that could be doubled performing one stage bilateral TKA [21, 22].

Results showed an average age under 60 years for THA and under 70 years for TKA. Taking into account the physiologic demands of patients undergoing simultaneous bilateral arthroplasty, previous studies have pointed out that they are generally younger, have lower BMI and less comorbidities than patients undergoing unilateral or staged bilateral arthroplasty [23–26].

Our results showed that the overall male/female ratio was almost equal for THA over the study period. It is known that after the age of 40, both sexes have an increasing risk of developing knee and hip OA, peaking at ages around 75–80 years [10, 27, 28]. The early and late peaks of arthroplasty female predominance from our results could be attributable to some common causes of secondary hip involvement that mainly affect women in these two different age groups. RA affects at least twice as many women as men [29], “pincer type” femoroacetabular impingement is seen more frequently in young adult women [30], and female sex is a well-known risk factor for developmental dysplasia of the hip [31, 32]. These are among the leading causes of secondary early hip OA requiring surgical treatment, often bilaterally. In contrast, the female predominance in the elderly could be linked to the longer women’s life expectancy and better general conditions to undergo a simultaneous bilateral THA. The present study showed that a minor part of bilateral one stage THA are performed to treat hip fractures: polytraumatized patients may require joint replacement for fracture treatment (sometimes bilaterally), partly justifying our results.

Over the study period, females represented at each year the majority of patients who underwent bilateral TKA in Italy. This is in line with data from other studies, indicating that nearly two thirds of knee arthroplasties are performed on women in the United States [33]. Many authors have investigated gender differences in the knee anatomy [34, 35]: women typically have a larger quadriceps angle (Q-angle) [36], a less pronounced anterior condyle [34], and a smaller mediolateral to anteroposterior aspect ratio [37]. It has led to the idea of gender-specific knee prostheses with controversial results [38].

A significant decrease in hospitalization days for both THA and TKA was noticed in Italy. Variations over time of the length of stay is likely to be the consequence of hospitals generally shortening hospitalization for economic reasons. Moreover, it could be due to the widespread adoption of fast-track arthroplasty protocols in developed countries. Fast-track hip and knee replacement aims to provide patients the best care possible creating a shortened approach from admission till discharge by combining evidence-based clinical and therapeutic procedures with organizational optimization [39]. The goal is to decrease morbidity and mortality, to quicken the achievement of early postoperative functional milestones, resulting in a shorter length of stay and a higher patient satisfaction [39]. As expected for the worse general clinical conditions, older patients needed on average more days of hospital stay.

Over the study period, primary bilateral hip and knee OA were the most common causes requiring one stage bilateral THA or TKA in Italy. These data underline the burden of OA, one of the most common chronic diseases today, that due to longer life expectancies is predicted to increase both its frequency and incidence [1].

Our study has some limitations. All of the reported diagnoses and procedures in this study were recorded using the ICD-9-CM, which is based on administrative data from different hospitals and Italian regions. Due to the numerous hospitals involved, it is difficult to identify diagnosis, procedure or coding errors. The ICD-9-CM allows for using multiple codes for the same surgical procedure. This heterogeneity in coding could lead to overestimate or underestimate our results. Because the ICD-9 coding was performed by surgeons, individual inter-observer variations are possible. A potential limitation is the lack of outcome scores. Moreover, the present study does not compare data from patients undergoing unilateral and bilateral procedures.

## Conclusions

The purpose of this study was to estimate demographic features, hospitalization trends and incidence of bilateral simultaneous THA and TKA in Italy by using hospitalization records as official information sources. Except for the 2001–2003 period, there was an increasing incidence trend of one stage bilateral THA and TKA in Italy. Although it still represents a matter of controversy, many studies have shown the potential advantages of one stage bilateral arthroplasty versus two stage. Results of the present study also highlighted the demographic features of patients undergoing bilateral one stage hip and knee arthroplasty (average age under 60 years for THA and under 70 years for TKA. This study also confirmed in the Italian population the role of primary hip and knee OA as a leading cause of bilateral joint replacement. Therefore, epidemiological studies may contribute to gather data for establishing international guidelines about the appropriate indications for one stage bilateral simultaneous THA or TKA versus two stage.

## Data Availability

The datasets used and/or analyzed during the current study are available from the corresponding author on reasonable request. The access to the database is on request. All data were obtained by the Direzione Generale della Programmazione Sanitaria—Banca Dati SDO of the Italian Ministry of Health.

## Abbreviations

TJA: total joint arthroplasty
TKA: total knee arthroplasty
THA: total hip arthroplasty
OA: osteoarthritis
RA: rheumatoid arthritis
SDO: the National Hospital Discharge records
ISTAT: the National Institute for Statistics
ICD-9-CM: the International Classification of Diseases, Ninth Revision, Clinical Modification

## References

[1] Pereira D, Ramos E, Branco J (2015). Osteoarthritis. Acta Med Port.

[2] Ethgen O, Bruyère O, Richy F, Dardennes C, Reginster JY (2004). Health-related quality of life in total hip and total knee arthroplasty. A qualitative and systematic review of the literature. J Bone Joint Surg Am.

[3] Longo UG, Ciuffreda M, Candela V, Berton A, Maffulli N, Denaro V (2019). Hip scores: a current concept review. Br Med Bull.

[4] Longo UG, De Salvatore S, Piergentili I, Indiveri A, Di Naro C, Santamaria G, Marchetti A, Marinis MG, Denaro V. Total hip arthroplasty: minimal clinically important difference and patient acceptable symptom state for the Forgotten Joint score 12. Int J Environ Res Public Health 2021, 18(5). 10.3390/ijerph18052267 PMC7956707 33668868

[5] Longo UG, De Salvatore S, Candela V, Berton A, Casciaro C, Sciotti G, Cirimele G, Marchetti A, Piergentili I, De Marinis MG et al. Unicompartmental knee arthroplasty: minimal important difference and patient acceptable symptom state for the Forgotten Joint score. Med (Kaunas) 2021, 57(4). 10.3390/medicina57040324 PMC8065647 33915704

[6] Sloan M, Premkumar A, Sheth NP (2018). Projected Volume of Primary Total Joint Arthroplasty in the U.S., 2014 to 2030. J Bone Joint Surg Am.

[7] Kurtz SM, Ong KL, Lau E, Bozic KJ (2014). Impact of the economic downturn on total joint replacement demand in the United States: updated projections to 2021. J Bone Joint Surg Am.

[8] Jaffe WL, Charnley J (1971). Bilateral Charnley low-friction arthroplasty as a single operative procedure. A report of fifty cases. Bull Hosp Joint Dis.

[9] Grauer JD, Cracchiolo A, Finerman GA, Dorey FJ (1986). Bilateral hip and knee arthroplasty. J Arthroplasty.

[10] Arden N, Nevitt MC (2006). Osteoarthritis: epidemiology. Best Pract Res Clin Rheumatol.

[11] Sobh AH, Siljander MP, Mells AJ, Koueiter DM, Moore DD, Karadsheh MS (2018). Cost analysis, complications, and Discharge Disposition Associated with simultaneous vs staged bilateral total knee arthroplasty. J Arthroplasty.

[12] Hutchinson JR, Parish EN, Cross MJ (2006). A comparison of bilateral uncemented total knee arthroplasty: simultaneous or staged?. J Bone Joint Surg Br.

[13] Urban MK, Chisholm M, Wukovits B (2006). Are postoperative complications more common with single-stage bilateral (SBTKR) than with unilateral knee arthroplasty: guidelines for patients scheduled for SBTKR. HSS J.

[14] Leonard L, Williamson DM, Ivory JP, Jennison C (2003). An evaluation of the safety and efficacy of simultaneous bilateral total knee arthroplasty. J Arthroplasty.

[15] Huang L, Xu T, Li P, Xu Y, Xia L, Zhao Z (2019). Comparison of mortality and complications between bilateral simultaneous and staged total hip arthroplasty: a systematic review and meta-analysis. Med (Baltim).

[16] Lin AC, Chao E, Yang CM, Wen HC, Ma HL, Lu TC (2014). Costs of staged versus simultaneous bilateral total knee arthroplasty: a population-based study of the Taiwanese National Health Insurance Database. J Orthop Surg Res.

[17] Bohm ER, Molodianovitsh K, Dragan A, Zhu N, Webster G, Masri B, Schemitsch E, Dunbar M (2016). Outcomes of unilateral and bilateral total knee arthroplasty in 238,373 patients. Acta Orthop.

[18] Papalia R, Vespasiani-Gentilucci U, Longo UG, Esposito C, Zampogna B, Antonelli Incalzi R, Denaro V (2019). Advances in management of periprosthetic joint infections: an historical prospective study. Eur Rev Med Pharmacol Sci.

[19] Kamath AF, Monteiro EL, Spranger A, Impellizzeri F, Leunig M (2016). Simultaneous versus staged bilateral direct anterior total hip arthroplasty: are early patient-centered outcomes equivalent?. Acta Orthop Belg.

[20] Gliklich RE, Dreyer NA, Leavy MB. Registries for Evaluating Patient Outcomes: A User’s Guide. In, edn.; 2014. 24945055

[21] Gurunathan U (2013). Perioperative considerations of bilateral total knee replacement: a review. J Clin Anesth.

[22] Khanna A, Gougoulias N, Longo UG, Maffulli N (2009). Minimally invasive total knee arthroplasty: a systematic review. Orthop Clin North Am.

[23] Morton JS, Kester BS, Eftekhary N, Vigdorchik J, Long WJ, Memtsoudis SG, Poultsides LA (2020). Thirty-day outcomes after bilateral total hip arthroplasty in a Nationwide Cohort. Arthroplast Today.

[24] Stavrakis AI, SooHoo NF, Lieberman JR (2015). Bilateral Total Hip Arthroplasty has similar complication rates to unilateral total hip arthroplasty. J Arthroplasty.

[25] Rasouli MR, Maltenfort MG, Ross D, Hozack WJ, Memtsoudis SG, Parvizi J (2014). Perioperative morbidity and mortality following bilateral total hip arthroplasty. J Arthroplasty.

[26] Poultsides LA, Triantafyllopoulos GK, Memtsoudis SG, Do HT, Alexiades MM, Sculco TP (2017). Perioperative Morbidity of same-day and staged bilateral total hip arthroplasty. J Arthroplasty.

[27] Oliveria SA, Felson DT, Reed JI, Cirillo PA, Walker AM (1995). Incidence of symptomatic hand, hip, and knee osteoarthritis among patients in a health maintenance organization. Arthritis Rheum.

[28] Muraki S, Akune T, Oka H, Ishimoto Y, Nagata K, Yoshida M, Tokimura F, Nakamura K, Kawaguchi H, Yoshimura N (2012). Incidence and risk factors for radiographic knee osteoarthritis and knee pain in Japanese men and women: a longitudinal population-based cohort study. Arthritis Rheum.

[29] van der Woude D, van der Helm-van Mil AHM (2018). Update on the epidemiology, risk factors, and disease outcomes of rheumatoid arthritis. Best Pract Res Clin Rheumatol.

[30] Ganz R, Parvizi J, Beck M, Leunig M, Nötzli H, Siebenrock KA (2003). Femoroacetabular impingement: a cause for osteoarthritis of the hip. Clin Orthop Relat Res.

[31] Yang S, Zusman N, Lieberman E, Goldstein RY. Developmental Dysplasia of the hip. Pediatrics 2019, 143(1). 10.1542/peds.2018-1147 30587534

[32] Jacobsen S, Sonne-Holm S (2005). Hip dysplasia: a significant risk factor for the development of hip osteoarthritis. A cross-sectional survey. Rheumatology (Oxford).

[33] Kurtz S, Ong K, Lau E, Mowat F, Halpern M (2007). Projections of primary and revision hip and knee arthroplasty in the United States from 2005 to 2030. J Bone Joint Surg Am.

[34] Conley S, Rosenberg A, Crowninshield R (2007). The female knee: anatomic variations. J Am Acad Orthop Surg.

[35] Zeng YM, Wang Y, Zhu ZA, Dai KR (2012). Effects of sex and lower extremity alignment on orientation of the knee joint line in knee surgery. Chin Med J (Engl).

[36] Woodland LH, Francis RS (1992). Parameters and comparisons of the quadriceps angle of college-aged men and women in the supine and standing positions. Am J Sports Med.

[37] Chin KR, Dalury DF, Zurakowski D, Scott RD (2002). Intraoperative measurements of male and female distal femurs during primary total knee arthroplasty. J Knee Surg.

[38] Cheng T, Zhu C, Wang J, Cheng M, Peng X, Wang Q, Zhang X (2014). No clinical benefit of gender-specific total knee arthroplasty. Acta Orthop.

[39] Husted H (2012). Fast-track hip and knee arthroplasty: clinical and organizational aspects. Acta Orthop Suppl.

